# Granular-Lids

**Published:** 1874-05

**Authors:** 


*Granular Lids.*—A number of cases of this kind have been successfully treated by the use of liq. plumbi subacetatis after all other ordinary means had failed. In one case ectropion was present in a marked degree, and with this treatment had almost entirely disappeared. About two drops of the undiluted liquor are dropped into the eye once in three or four days. It is important that the remedy should not be applied too often, lest the irritation produced should be continued, and prevent the beneficial effects which may follow if used at somewhat lengthy intervals.

Give the lids an opportunity to get better after each application.

